# Unmasking Hirata: a mysterious case of Hypoglycemia triggered by immunologic storm

**DOI:** 10.1093/omcr/omaf289

**Published:** 2026-01-25

**Authors:** Mohammed AbuBaha, Hossam Salameh, Bara AbuBaha, Yasmin Dahabreh, Omar Marouf, Mousa Atary, Heba Qubaja, Amal Mansor, Hatem M Taha

**Affiliations:** Department of Medicine, An-Najah National University, Rafidia street, Nablus P400, Palestine; Department of Medicine, An-Najah National University, Rafidia street, Nablus P400, Palestine; Department of Medicine, An-Najah National University, Rafidia street, Nablus P400, Palestine; Department of Medicine, An-Najah National University, Rafidia street, Nablus P400, Palestine; Department of Internal Medicine, Palestine Medical Complex, Al Ma'ahed street, Ramallah 601, Palestine; Institute of Community and Public Health, Birzeit University, Al Marj street, Birzeit 627, Palestine; Department of Medicine, An-Najah National University, Rafidia street, Nablus P400, Palestine; Department of Medicine, An-Najah National University, Rafidia street, Nablus P400, Palestine; Head of Medical Departments, Palestine Medical Complex, Al Ma'ahed street, Ramallah 601, Palestine; Faculty of Medicine and Health Sciences, An-Najah National University, Rafidia street, Nablus P400, Palestine

**Keywords:** Insulin Autoimmune Syndrome, Hirata disease, hypoglycemia, Autoimmunity

## Abstract

We report a rare case of Insulin Autoimmune Syndrome in a 35-year-old woman with prior autoimmune features who developed hypoglycemia after an allergic reaction. Diagnosis was confirmed by insulin autoantibodies. Immunosuppressive therapy yielded significant improvement. This case suggests a broader autoimmune process may underlie seemingly isolated IAS presentations.

## Introduction

Hirata syndrome, also known as insulin autoimmune syndrome (IAS), is a rare disorder characterized by hyperinsulinemic hypoglycemia that occurs independently due to elevated serum levels of insulin autoantibodies (IAA), which is commonly associated with HLA-DR4 (DRB1^*^0406) [1].

The first case of IAS was reported in 1970 [2], and since then, 197 IAS cases from 1970 to 1992 have been documented. By the end of 2007, 325 patients’ records from the first and second nationwide surveys on sudden hypoglycemia were acquired [3]. IAS is considered the third leading cause of spontaneous hypoglycemia in Japan [3],

Another significant finding is that a person’s ethnic origin has a substantial impact on their tendency to develop IAS, with East Asians showing a higher incidence than Caucasians, which can be explained because Caucasians have a low prevalence of (DRB1^*^0406) [3].

## Case presentation

A 35-year-old previously healthy woman initially presented with symptoms consistent with carpal tunnel syndrome and was managed with alpha-lipoic acid in December of 2023. Subsequently, 10 days later, the patient was exposed to a lime scale removal cleaning product, and the patient developed an acute allergic reaction characterized by pruritic urticarial rash, erythema, and facial flushing within hours of exposure, without airway compromise, angioedema, or hypotension.

The patient was managed initially with loratadine, an antihistamine drug, 10 mg for one day, which was ineffective, and her symptoms persisted. After that, she was switched to a two-week course of bilastine, a second-generation H1 antihistamine 20 mg orally once daily for 14 days, which led to a significant improvement in her allergic symptoms. However, three days after initiating bilastine, the patient began experiencing new-onset dizziness, profuse sweating, tachycardia, and shortness of breath; at the same time, the patient experienced an episode of seizure during her sleep (according to whom, a witness?). After two weeks, episodes grew more frequent and severe, and because of that, she returned to her physician. Laboratory evaluation revealed fasting hypoglycemia, which was confirmed to be profoundly low on multiple occasions. Specific documented values were reported at 38 mg/dL, 41 mg/dL on this visit (Normal range: 70–100 mg/dL), accompanied by concurrent serum fasting insulin levels that were exceptionally high, far exceeding the normal range of 2.6–24.9 μU/mL, with documented levels were > 2778 μU/mL and C-peptide of 5.04 ng/mL (reference: 0.78–5.19 ng/mL), confirming endogenous insulin. A postprandial glucose was checked and revealed recurrent values ranging 34–58 mg/dL, with the lowest value at 34 mg/dL, associated with neuroglycopenic symptoms including blurry vision, loss of consciousness, and tonic–clonic seizures An additional confirmatory autoimmune markers workup was done, which revealed a high titer of Anti-Insulin Antibodies at 43.0 U/ml (Normal: < 10 U/ml). A systematic and thorough investigation was conducted to rule out other potential causes of hyperinsulinemic hypoglycemia, which included a pancreatic MRI that showed no evidence of a mass or lesion to rule out the primary differential diagnosis of an insulin-secreting pancreatic tumor. Moreover, a systematic and comprehensive autoimmune panel was performed. Investigations for systemic lupus erythematosus (SLE) were negative, with a negative Anti-Nuclear Antibody (ANA) and Anti-nDNA antibody test on the Extractable Nuclear Antigens (ENA) panel, which screens for various connective tissue diseases, was also negative, except for a single positive PO antibody, whose clinical significance was considered low in this context. Rheumatoid Factor (RF) was negative (< 20.0 IU/mL) with negative CRP and normal ESR (7 mm/hr) within the normal values (0–20 mm/hr), making rheumatoid arthritis unlikely. Endocrine and Thyroid Autoimmunity were also done. Thyroid-stimulating hormone (TSH) levels were consistently within the normal range across multiple tests, indicating that the patient was clinically euthyroid. Her level was 1.9026 μU/mL, within the normal range (0.35–4.94 μU/mL) on this last visit. Interestingly, the Thyroid Stimulating Immunoglobulin (TSI) test returned a borderline positive result of 1.4 IU/L (Negative: < 1.22 IU/L). However, with normal TSH levels and negative Anti-Thyroid Peroxidase (TPO) antibodies, this was interpreted as evidence of subclinical thyroid autoimmunity without active Graves’ disease. The patient’s Parathyroid Hormone (PTH) level was 71.1 pg/mL, which is elevated above the provided normal reference range of 15–68 pg/mL. This finding is significant and requires careful interpretation, especially in the context of her other lab values. Given that cortisol deficiency can cause hypoglycemia, the patient’s morning cortisol level was 10.20 μg/dL (Normal range: 3.7–19.4 μg/dL), and the concurrent Adrenocorticotropic Hormone (ACTH) level was 25.3 pg/mL (Normal range: 7.4–64.3 pg/mL). These expected results effectively ruled out both primary and secondary adrenal insufficiency as a cause for her hypoglycemic episodes. Anti-glutamic acid decarboxylase (GAD) (<0.24 IU/mL) and 21-hydroxylase antibodies (5.71 U/ml; negative: < 10) were also negative, reducing the likelihood of concurrent Type 1 Diabetes or autoimmune adrenal disease, respectively. Throughout her diagnostic journey, the patient exhibited several significant but fluctuating abnormalities in her complete blood count (CBC). The patient’s laboratory results revealed several notable abnormalities in her blood counts. She presented with a mild, fluctuating anemia, with her hemoglobin (Hb) level dipping to as low as 11.5 g/dL on multiple occasions. This anemia was further characterized as distinctly microcytic and hypochromic, with a consistently low mean corpuscular Volume (MCV) ranging from 74.5 to 78.2 fL, and correspondingly low mean corpuscular hemoglobin (MCH) and MCHC values. This was accompanied by significant shifts in her white blood cell differential, as shown in Table 1 below.

**Table 1:** CBC results with WBC differential results.

Parameter	Patient Values	Reference Range
Hemoglobin (Hb)	11.5 g/dL	12–16 g/dL
Mean Corpuscular Volume (MCV)	74.5–78.2 fL	80–96 fL
Eosinophils (%)	0.3–0.4%	1–3%
Monocytes (%)	0.5–2.5%	3–7%
Basophils (%)	0.9–1.4%	0–0.75%

Given the combination of severe hypoglycemia, markedly elevated insulin, normal C-peptide, and high insulin autoantibodies, a diagnosis of Insulin Autoimmune Syndrome (IAS) was confirmed. The patient underwent a 1-week home blood glucose monitoring to support the diagnosis, which involved adhering to a high-complex-carbohydrate diet and discontinuing bilastine. The list of differentials is demonstrated in Table 2 below, with Table 3 showing various test results.

**Table 2:** List of differentials and insights.

Suspected Cause	Rationale for exclusion
Insulinoma	No tumor seen on MRI.
Self-induced Hypoglycemia	No history of insulin abuse; antibodies confirmed IAS.
Adrenal insufficiency	Normal morning cortisol (10.2 μg/dL) and ACTH levels; no clinical features of adrenal failure.
Non-insulinoma pancreatogeneous hypoglycemia syndrome	Hypoglycemia occurred during fasting, not solely postprandial; no diffuse pancreatic abnormality; insulin autoantibodies explain biochemical profile.
Paraneoplastic syndrome (IGF-II–secreting tumor)	Such tumors cause hypoglycemia with low insulin/C-peptide; here insulin was markedly elevated; no tumor identified on imaging.
Severe Infection	No fever or signs of illness or infection.

**Table 3:** Laboratory results.

Test	Patient Result	Reference Range
Glucose/Insulin		
Fasting Plasma Glucose	38–41 mg/dL	70–100 mg/dL
Postprandial Glucose	34–58 mg/dL	70–140 mg/dL
Serum Insulin	>2778 μU/mL	2.6–24.9 μU/mL
C-peptide	5.04 ng/mL	0.78–5.19 ng/mL
Anti-Insulin Antibodies	43 U/mL	<10 U/mL
Endocrine Function		
Morning Cortisol	10.2 μg/dL	3.7–19.4 μg/dL
ACTH	25.3 pg/mL	7.4–64.3 pg/mL
TSH	1.9026 μU/mL	0.35–4.94 μU/mL
Thyroid Stimulating Immunoglobulin (TSI)	1.4 IU/L	<1.22 IU/L
Anti-TPO antibodies	Negative	<35 IU/mL
Parathyroid Hormone (PTH)	71.1 pg/mL	15–68 pg/mL
Autoimmune Markers		
ANA	Negative	Negative
Anti-dsDNA	Negative	Negative
ENA panel	Negative (except single PO antibody)	Negative
Rheumatoid Factor (RF)	<20 IU/mL	<20 IU/mL
CRP	Normal	<5 mg/L
ESR	7 mm/hr	0–20 mm/hr
Anti-GAD antibodies	<0.24 IU/mL	<1 IU/mL
21-hydroxylase antibodies	5.71 U/mL	<10 U/mL

**Table 4:** Literature review.

Author (Year)	Country	Suspected trigger	Hypoglycemia pattern	Peak insulin (μU/mL)	Anti-insulin Ab	HLA (if reported)	Treatment	Outcome
**Cappellani (2018)** [22]	Italy	Alpha-lipoic acid (ALA)	Mixed/unspecified	2465	Positive (530 U/mL)	DRB1^*^04, ^*^15	Dietary modification; drug withdrawal	Spontaneous remission
**Rajpal (2017)** [23]	USA	Clopidogrel (sulfhydryl metabolite)	Fasting & post-absorptive	40 000	Positive (59.3 nmol/L)^*^	DRB1^*^0404	Stop clopidogrel; glucocorticoids	Remission
**Roh (2013) – Case 1** [24]	Korea	Methimazole	Fasting	1268	Positive (titer high)	DRB1^*^0406	Stop methimazole; diet	Resolution < 1 month
**Roh (2013)—Case 2** [24]	Korea	Methimazole	Fasting	>3000	Positive (titer high)	DRB1^*^0406	Stop methimazole; diet	Resolution < 1 month
**Abeyagunawardena (2024)** [25]	Sri Lanka	Omeprazole and spice mix (suspected)	Fasting	>6000	Positive (>300 U/mL)	NR	Drug cessation (reported); other NR	NA
**Saxon (2016)** [26]	USA	None reported	Severe, refractory (mixed)	3641	Positive (high titer)	DR4 associated (not genotyped)	Rituximab ×2 + CGM	Remission
**okumura (2024)** [27]	Japan	None	Post-prandial predominant	86.3	3201	HLA-DR4.	frequent and small meals	

Prednisolone was then initiated at 35–40 mg daily in mid-February 2025, just prior to Ramadan (March 10, 2025), to address severe hypoglycemia due to IAS. After approximately 10 days, azathioprine (AZA) was added to provide steroid-sparing immunosuppression, and prednisolone was reduced to 30 mg daily for 5 weeks. Prednisolone was then gradually tapered over the subsequent 2 months by 10 mg every 2 weeks until reaching 10 mg every other day, after which it was discontinued. Azathioprine was maintained at 50 mg daily during the tapering of prednisolone and subsequently stopped as the patient’s clinical condition improved. This six-month immunosuppressive regimen resulted in substantial clinical improvement and a reduction in insulin levels from approximately 2778 μU/mL to around 100 μU/mL (normal 2.6–24.9 μU/mL).

## Discussion

Insulin Autoimmune Syndrome (IAS), first described by Dr. Hirata in 1970 [4], is a rare but significant cause of spontaneous, non-fasting (reactive) hypoglycemia characterized by hyperinsulinemic hypoglycemia in individuals without prior exposure to insulin or an insulinoma, and defined by very high titers of polyclonal insulin autoantibodies (IAA) [5].

The pathophysiology involves genetic susceptibility, most strongly linked to HLA-DRB1*04:06 in* Japanese patients*,* and HLA-DRB104:03 and 104:07 in Caucasians, which allows efficient presentation of insulin peptides to T cells [6], combined with environmental triggers, most notably sulfhydryl-containing compounds such as alpha-lipoic acid, methimazole, carbimazole, penicillamine, captopril, hydralazine, and interferon-alpha, which can modify insulin’s disulfide bonds, rendering it immunogenic [1, 7, 8].

Autoantibodies bind to postprandial insulin, initially sequestering it and causing transient hyperglycemia. This is followed by unpredictable dissociation several hours later, resulting in a sudden surge of free insulin at a time when glucose levels are already falling, which can lead to severe hypoglycemia [1].

Clinically, IAS presents with reactive hypoglycemia 3–5 hours post-meal (rarely fasting), with neuroglycopenic symptoms (confusion, dizziness, visual disturbance, seizures, loss of consciousness) and autonomic features (sweating, palpitations, tremor, anxiety) [9]. Diagnosis requires Whipple’s triad [10], characterized by extremely high serum insulin levels (often exceeding 100 μU/mL) [11], standard/elevated C-peptide (indicating endogenous insulin production) [12], and the hallmark detection of high-titer insulin autoantibodies [13].

Variations in cases reported in the literature are addressed and demonstrated in Table 4.

This patient hypoglycemia presented with both fasting and postprandial episodes, with glucose values as low as 34 mg/dL postprandially and 38 mg/dL fasting, associated with neuroglycopenic symptoms including dizziness, blurry vision, seizures, and loss of consciousness. Serum insulin levels were markedly elevated at 2778 μU/mL, while C-peptide levels were normal (5.04 ng/mL), and anti-insulin antibodies were high (43 U/mL), confirming endogenous hyperinsulinemia due to IAS. However, structural causes such as an insulinoma were ruled out by a normal pancreatic MRI. Although she had prior alpha-lipoic acid exposure over a year before symptom onset. It is recognized that Hirata syndrome can occur 30–120 days after starting the medication, and in rare cases, a longer latency period is possible; therefore, a contribution from prior alpha-lipoic acid cannot be completely excluded, with the temporal association with an acute allergic reaction to environmental exposure and initiation of bilastine may have transiently enhanced immune activation (GREAT LINE). However, causality cannot be definitively established. Supporting her predisposition to autoimmunity, thyroid evaluation revealed normal TSH (1.90 μU/mL) with borderline positive TSI (1.4 IU/L) and negative anti-TPO antibodies, suggesting subclinical thyroid autoimmunity without active Graves’ disease. Hematologic testing showed mild, fluctuating microcytic anemia, persistent eosinopenia (0.3–0.4%, normal 1–3%), monocytopenia (0.5–2.5%, normal 3–7%), intermittent basophilia (0.9–1.4%), normal < 0.75%) and thrombocytopenia, indicating variable immune or inflammatory activity. Rheumatoid arthritis was ruled out based on negative rheumatoid factor (RF < 20 IU/mL), normal CRP, and normal ESR (7 mm/hr). Her persistently raised PTH with normal calcium and vitamin D likely reflects either secondary hyperparathyroidism (compensatory/early), physiological fluctuation, or normocalcemic primary hyperparathyroidism. It appears to be an incidental and unrelated finding rather than autoimmune hyperparathyroidism, which is uncommon, though follow-up is warranted to exclude evolving parathyroid pathology. Together, these findings suggest a background of immune dysregulation that may have predisposed her to IAS, with both drug exposure and allergic response potentially serving as temporary triggers. Immunosuppressive therapy with prednisolone and azathioprine successfully reduced insulin levels and improved hypoglycemic episodes, highlighting the autoimmune etiology.

Insulin Autoimmune Syndrome (IAS) requires a methodical, customised approach to management. Although spontaneous remission is also possible, the first approach is to identify and stop any potential triggering medications, such as those that contain sulfhydryl [1].

To identify and stop hypoglycemia episodes, it is essential to monitor blood sugar levels. Small, frequent meals with low-glycemic carbs, or alpha-glucosidase inhibitors (like acarbose) are recommended [14].

While intravenous glucose is typically used only for severe or nocturnal hypoglycemia, pharmacologic therapy may employ somatostatin analogues or diazoxide in certain situations to reduce insulin secretion [15, 16]. For severe or chronic illness, immunosuppressive therapy is recommended. High-dose corticosteroids are frequently used as a first line of treatment [17], while steroid-sparing medications such as azathioprine are taken into consideration for longer-term care [18].

To lessen or eradicate insulin autoantibodies, rituximab, a B-cell-depleting monoclonal antibody, has been used in refractory cases, occasionally in conjunction with immunoadsorption [19, 20]. Additionally, plasmapheresis may also be used in severe cases to quickly reduce antibody titers in cases of severe, acute hypoglycemia [21].

## Conclusion

This case highlights a rare, possibly immune-triggered form of IAS that arose shortly after an allergic episode in a patient with preexisting autoimmune markers and systemic immune abnormalities. The presentation raises an important question: Is this truly isolated IAS, or is it the initial manifestation of a broader autoimmune syndrome? Further investigations, autoimmune panels, and close longitudinal follow-up are warranted. The absence of C-peptide measurement remains a critical limitation, underscoring the need for a comprehensive metabolic workup in suspected IAS.

## References

[1] Cappellani D, Macchia E, Falorni A. et al. Insulin autoimmune syndrome (Hirata disease): a comprehensive review fifty years after its first description. Diabetes Metab Syndr Obes 2020;13:963–78. 10.2147/DMSO.S219438 32308449 PMC7136665

[2] Yuk H, Tominaga M, Ito JI. et al. Spontaneous Hypoglycemia with insulin autoimmunity in graves’ disease. Ann Intern Med 1974;81:214–8. 10.7326/0003-4819-81-2-214 4407888

[3] Uchigata Y, Hirata Y, Iwamoto Y. Insulin autoimmune syndrome (Hirata disease): epidemiology in Asia, including Japan. Diabetol Int 2010;1:21–5. 10.1007/s13340-010-0001-z

[4] Hirata Y, Ishizu H, Ouchi N. Insulin autoimmunity in a case of spontaneous hypoglycemia. J Jpn Diabetes Soc 1970;13:312–20.

[5] Ishizuka T, Ogawa S, Mori T. et al. Characteristics of the antibodies of two patients who developed daytime hyperglycemia and morning hypoglycemia because of insulin antibodies. Diabetes Res Clin Pract 2009;84:e21–3. 10.1016/j.diabres.2009.02.007 19328577

[6] Uchigata Y, Hirata Y, Omori Y. et al. Worldwide differences in the incidence of insulin autoimmune syndrome (Hirata disease) with respect to the evolution of HLA-DR4 alleles. Hum Immunol 2000;61:154–7. 10.1016/S0198-8859(99)00144-5 10717808

[7] Hirata Y . Methimazole and insulin autoimmune syndrome with hypoglycemia. Lancet 1983;322:1037–8. 10.1016/s0140-6736(83)91031-0 6138590

[8] Uchigata Y . The novel agent, alpha lipoic acid, can cause the development of insulin autoimmune syndrome. Intern Med 2007;46:1321–2. 10.2169/internalmedicine.46.0221 17827827

[9] Martens P, Tits J. Approach to the patient with spontaneous hypoglycemia. Eur J Intern Med 2014;25:415–21. 10.1016/j.ejim.2014.02.011 24641805

[10] International Hypoglycaemia Study G . Glucose concentrations of less than 3.0 mmol/L (54 mg/dL) should be reported in clinical trials: a joint position statement of the american diabetes association and the european association for the study of diabetes. Diabetes Care 2017;40:155–7. 10.2337/dc16-2215 27872155

[11] Virally ML, Timsit J, Chanson P. et al. Insulin autoimmune syndrome: a rare cause of hypoglycaemia not to be overlooked. Diabetes Metab 1999;25:429–31. 10592867

[12] Cryer PE, Axelrod L, Grossman AB. et al. Evaluation and management of adult hypoglycemic disorders: an Endocrine Society clinical practice guideline. J Clin Endocrinol Metab 2009;94:709–28. 10.1210/jc.2008-1410 19088155

[13] Ismail AA . Testing for insulin antibodies is mandatory in the differential diagnosis of hypoglycaemia in nondiabetic subjects. Clin Endocrinol 2012;76:603–4. 10.1111/j.1365-2265.2011.04259.x 21995821

[14] Redmon JB, Nuttall FQ. Autoimmune hypoglycemia. Endocrinol Metab Clin N Am 1999;28:603–18 vii. 10.1016/S0889-8529(05)70090-6 10500933

[15] Jain N, Savani M, Agarwal M. et al. Methimazole-induced insulin autoimmune syndrome. Ther Adv Endocrinol Metab 2016;7:178–81. 10.1177/2042018816658396 27540463 PMC4973408

[16] Church D, Cardoso L, Kay RG. et al. Assessment and management of anti-insulin autoantibodies in varying presentations of insulin autoimmune syndrome. J Clin Endocrinol Metab 2018;103:3845–55. 10.1210/jc.2018-00972 30085133 PMC6179165

[17] Lupsa BC, Chong AY, Cochran EK. et al. Autoimmune forms of hypoglycemia. Medicine (Baltimore) 2009;88:141–53. 10.1097/MD.0b013e3181a5b42e 19440117

[18] Panikar V, Joshi S, Vadgama J. et al. Autoimmune hypoglycemia relapse on glucocorticoids, effectively treated with azathioprine. J Assoc Physicians India 2018;66:78–9. 31313558

[19] Church D, Hunter RW, Lyall M. et al. Resolution of Hypoglycemia and cardiovascular dysfunction after rituximab treatment of insulin autoimmune syndrome. Diabetes Care 2017;40:e80–2. 10.2337/dc17-0264 28461453

[20] Kroemer TM, Erler A, Tsourdi E. et al. Immunoadsorption followed by rituximab as a definitive treatment for insulin autoimmune syndrome (Hirata syndrome): a case report. Diabetes Care 2018;41:e23–4. 10.2337/dc17-1974 29282202

[21] Yaturu S, DePrisco C, Lurie A. Severe autoimmune hypoglycemia with insulin antibodies necessitating plasmapheresis. Endocr Pract 2004;10:49–54. 10.4158/EP.10.1.49 15251622

[22] Cappellani D, Sardella C, Campopiano MC. et al. Spontaneously remitting insulin autoimmune syndrome in a patient taking alpha-lipoic acid. Endocrinol Diabetes Metab Case Rep 2018;2018:18–0122. 10.1530/EDM-18-0122 PMC6300855 30532997

[23] Rajpal A, Kassem LS, Moscoso-Cordero M. et al. Clopidogrel-induced insulin autoimmune syndrome: a newly recognized cause of Hypoglycemia in a patient without diabetes. J Endocr Soc 2017;1:1217–23. 10.1210/js.2017-00316 29264578 PMC5686698

[24] Roh E, Kim YA, Ku EJ. et al. Two cases of Methimazole-induced insulin autoimmune syndrome in graves’ disease. Endocrinol Metab 2013;28:55–60. 10.3803/EnM.2013.28.1.55 PMC3811805 24396652

[25] Abeyagunawardena I, Madurapperuma C, Thuvarakan P. et al. Uncommon triggers of insulin autoimmune syndrome: a case report. J Med Case Rep 2024;18:292. 10.1186/s13256-024-04616-x 38926797 PMC11210015

[26] Saxon DR, McDermott MT, Michels AW. Novel Management of Insulin Autoimmune Syndrome with rituximab and continuous glucose monitoring. J Clin Endocrinol Metab 2016;101:1931–4. 10.1210/jc.2016-1097 26982011 PMC12119054

[27] Okumura H, Inaba S, Kawashima A. et al. Insulin autoimmune syndrome: a case report highlighting diagnostic pitfalls. Cureus. 2024;16:e64130. 10.7759/cureus.64130 39119412 PMC11307240

